# Behavioral and neural plasticity caused by early social experiences: the case of the honeybee

**DOI:** 10.3389/fphys.2013.00041

**Published:** 2013-08-23

**Authors:** Andrés Arenas, Gabriela P. Ramírez, María Sol Balbuena, Walter M. Farina

**Affiliations:** Grupo de Estudio de Insectos Sociales, Departamento de Biodiversidad y Biología Experimental, Facultad de Ciencias Exactas y Naturales, IFIBYNE-CONICET, Universidad de Buenos AiresBuenos Aires, Argentina

**Keywords:** behavior, honeybee, olfaction, plasticity, early experiences, associative learning

## Abstract

Cognitive experiences during the early stages of life play an important role in shaping future behavior. Behavioral and neural long-term changes after early sensory and associative experiences have been recently reported in the honeybee. This invertebrate is an excellent model for assessing the role of precocious experiences on later behavior due to its extraordinarily tuned division of labor based on age polyethism. These studies are mainly focused on the role and importance of experiences occurred during the first days of the adult lifespan, their impact on foraging decisions, and their contribution to coordinate food gathering. Odor-rewarded experiences during the first days of honeybee adulthood alter the responsiveness to sucrose, making young hive bees more sensitive to assess gustatory features about the nectar brought back to the hive and affecting the dynamic of the food transfers and the propagation of food-related information within the colony. Early olfactory experiences lead to stable and long-term associative memories that can be successfully recalled after many days, even at foraging ages. Also they improve memorizing of new associative learning events later in life. The establishment of early memories promotes stable reorganization of the olfactory circuits inducing structural and functional changes in the antennal lobe (AL). Early rewarded experiences have relevant consequences at the social level too, biasing dance and trophallaxis partner choice and affecting recruitment. Here, we revised recent results in bees' physiology, behavior, and sociobiology to depict how the early experiences affect their cognition abilities and neural-related circuits.

## Framework

Experiences at early stages of animals' life can shape later behavior in a dramatically and sometimes irreversible way. From the most extreme example of imprinting describing the attachment behaviors of geese soon after they hatched (Lorenz, 1935), a great range of sensory and cognitive experiences have been reported to play a role in shaping future behavior in many groups, including humans (Neal, 1972; Cornwell-Jones et al., 1988; Cramer et al., 1988; Gschanes et al., 1998; Matthews and Robbins, 2003; Pryce and Feldon, 2003; Schäble et al., 2007). In the last three decades, the honeybee *Apis mellifera* has been considered a model within the invertebrates to study the behavioral and neural plasticity caused by early experiences (Masson and Arnold, 1984, 1987; Winnington et al., 1996; Sigg et al., 1997; Farris et al., 2001; Brown et al., 2004). There are several reasons to justify this choice. First, honeybees exhibit an excellent predisposition to learn and retain neutral stimuli. Second, they have a relatively simple and accessible brain. Third, it is possible to manipulate the early experience of honeybees by assessing responses under controlled conditions. Finally and probably the most important reason is the changing behavioral contexts at which they are exposed during the adult lifespan.

Honeybees undergo an age-related polyethism which plays an important role in task allocation and division of labor within their colonies (Wilson, 1971; Michener, 1974). Newly emerged bees of the worker caste mainly clean the comb cells and care for brood inside the nest, while middle age bees process and store food until they initiate foraging from the third week of the adult life (Rösch, 1925; Lindauer, 1952; Seeley, 1982). This dynamic makes honeybees ideal models to analyze the effect of particular sensory stimuli during young adulthood on later behavior. Division of labor occurs because individuals differ in their preferred response. In a honeybee hive, nest-mates of different ages display different response thresholds to given stimuli, which enable the display of specific behaviors (Robinson, 1992; Page et al., 1998). The transition from one task to the next requires major changes in sensory and cognitive abilities which are accompanied by morphological and physiological changes in the brain (Fahrbach, 2006). Shift from in-hive tasks to foraging involves the development of a series of new and integrated skills such as flight navigation, food location and communication of information to the rest of the colony. At foraging ages the neural pathways are fully developed (Masson and Arnold, 1987; Winnington et al., 1996), providing bees with abilities to learn and retain relevant environmental stimuli. Foraging bees can, for example, learn floral odors while visiting rewarding flowers. Learning of olfactory cues can lead to memories that are stored in different neural substrates of the brain (Galizia et al., 2012; Giurfa and Sandoz, 2012; Menzel, 2012) and guide the foraging bee toward the learned stimuli (Dukas, 2008).

Whenever a successful forager returns to the hive, it searches for nest mates to share the collected liquid food that is transferred via mouth-to-mouth trophallaxis. As foragers may carry the scent of the flowers diluted in the nectar inside its crop, sharing of scented food allows different worker groups, even those not directly involved in foraging-related activities like nurse bees, to learn the nectar scent and gain a key information about the currently exploited food source (Pankiw et al., 2004; Grüter et al., 2006). Operational cast in charge of unloading nectar (receivers) is mainly comprised of middle age workers. They can accept or refuse to unload nectar mainly based on its quality (gustatory cues) and the quality of the alternative sources that are currently exploited in the field (Seeley, 1989). If the incoming nectar is too diluted, receivers may refuse to unload it, a decision that affects food distribution through the nest together with the spread of olfactory and gustatory information (Ramírez et al., 2010).

According to the properties of the exploited floral patch and the food storing level of the colony, successful foragers can result stimulated to perform recruiting dances (von Frisch, 1967). This signal communicates among other aspects the location of profitable food sources encoded in the dancers' maneuvers (von Frisch, 1967; Dyer, 2002; Riley et al., 2005; Grüter and Farina, 2009). Several bees can simultaneously follow a dancer, including experienced foragers that can be reactivated to collect resources (Biesmejer and Seeley, 2005; Grüter et al., 2008) or novice foragers that search for reliable information to initiate their activities (von Frisch, 1967; Riley et al., 2005).

As we can see, honeybees display a rich and interesting behavioral repertoire, in which thresholds of response and associative learning play a fundamental role in the framework of foraging activities. Several protocols have been developed to address these two main plastic components that influence the honeybees' decisions. Taking advantages of the fact that honeybees extend their proboscises as a reflex response to antennal stimulation with a sufficiently concentrated sucrose solution (Kuwabara, 1957; Takeda, 1961; Bitterman et al., 1983), their response threshold to sugar can be approximate by the lowest concentration that elicits the extension of the proboscis within successive presentations of increasing sucrose solutions (Page et al., 1998; Pankiw and Page, 1999). Olfactory memories can also be quantified using the proboscis extension response (PER), as bees that have associated a conditioned odor with a nectar or pollen reward protrude the proboscis when that stimulus is delivered onto the antennae (Gerber et al., 1996; Sandoz et al., 2000).

Although many studies have focused on sensory and cognitive capabilities of foraging age honeybees, very little is known about the role and importance of early experiences in the development of these abilities, their impact on long-lasting foraging decisions and their eventual contribution to coordinate complex tasks at a social scale. Some recent reports addressed the question about how gustatory and olfactory information acquired early in the adult life modifies the honeybee underlying individual and social behavior as well as their concomitant neurobiological processes and substrates. In this review we center on these new evidences that focused on the honeybee foraging-related behavior.

## Changes in gustatory responsiveness in young honeybees after the incoming of scented food

Honeybees assess the value of a nectar source according to their own perception (Scheiner et al., 1999, 2001) deciding, in turn, whether to forage on it or not. We know now that gustatory responsiveness rests on genetic bases (Page et al., 1998); however it can also be modulated by the environment (Pankiw et al., 2004; Martinez and Farina, 2008). Under controlled conditions, honeybees offered to forage high-concentration of a sucrose solution for 24 h presented higher thresholds to sucrose than those fed with low concentrated solutions (Pankiw et al., 2001, 2004), suggesting that previous foraging experiences can modulate the thresholds of this behavioral response in the short-term. Not only foragers, but pre-foraging bees are also able to adjust their sucrose response thresholds to sucrose. Qualitative changes in incoming nectar affect the behavior of 3–6 day-old members of the colony (Pankiw et al., 2004). Moreover, recent evidence indicates that receivers (pre-foraging bees of about 14 days of age) modify their sucrose response thresholds according to the quality of the food previously passed by the returning foragers (Martinez and Farina, 2008).

More experiments have been done to understand the role the young pre-foragers play in the propagation of gustatory and olfactory information of the food within the honeybee colony. Workers of the same age maintained under controlled conditions in the lab were subjected to different reward programs that changed in food quality. This experiment showed that 14-day-old bees have a higher modulation to adjust their response than bees of 7 days. Moreover, this study suggests that gustatory responsiveness of pre-foraging workers varied with the presence of a scent in the food. Interesting, workers that showed PER toward the odor that had been previously offered in their food (conditioned response, CR) presented higher responsiveness than those bees that did not extent their proboscises (Ramírez et al., 2010). Presence of stable memories and changes in gustatory responsiveness (measured as Gustatory Response Scores, i.e., GRSs) in middle age bees motivates the idea that olfactory memories could affect the sensitization to sugar (Ramírez et al., 2010). In the laboratory it has been described that immediately after a single association between the odor (as conditioned stimulus) and the sugar reward (as unconditioned stimulus) the learning process is dominated by a sensitization component (Menzel, 1999). Then, during paring the stimuli, sensitization to sugar could influence gustatory responsiveness by lowering the response thresholds of the conditioned bees.

**Figure 1:**
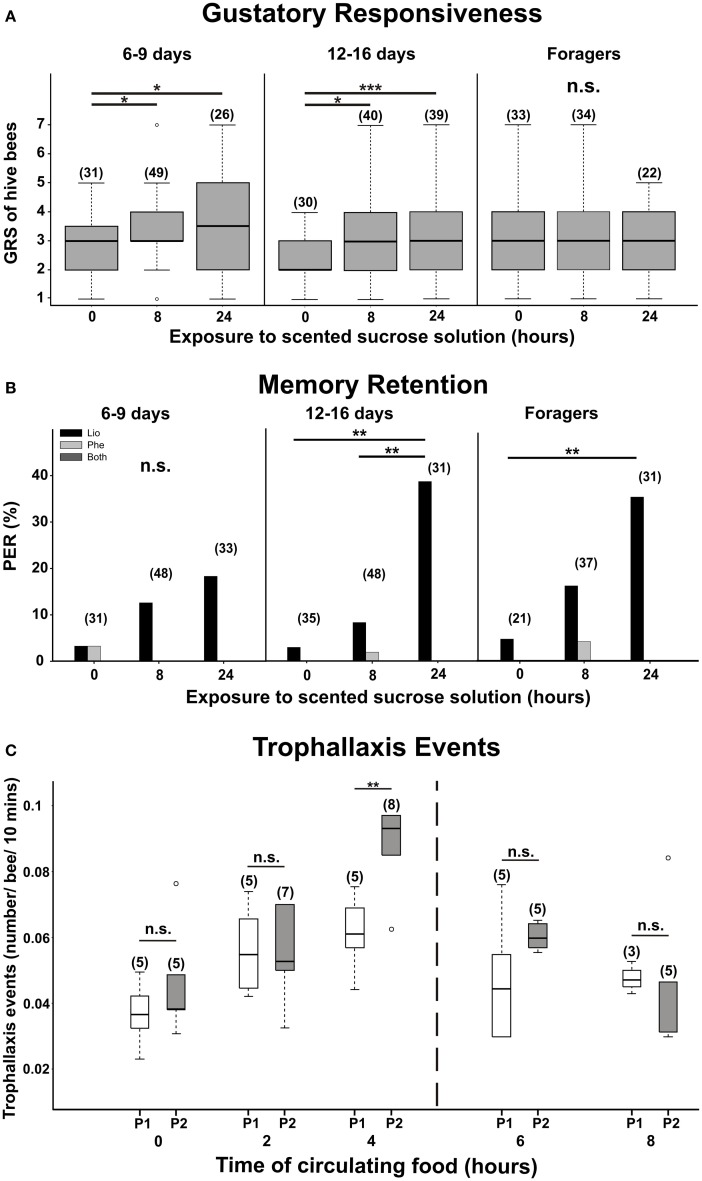
**Gustatory responsiveness and memory retention of free-flying bees after the influx of scented sucrose solution.** Bees of different age groups: 6/9 days old, 12/16 days old, and foragers were captured from the hive. Gustatory response scores (GRSs) **(A)** and proboscis extension responses (PER) **(B)** to the odor solution (LIO, black bars), a novel test odor (phenylacetaldehyde, PHE, gray bars), or both (dark gray bars) were measured during the incoming of unscented sucrose solution (15% w/w) (0 h) or after 8 h and 24 h of foraging on a scented sucrose solution (linalool, LIO, 15% w/w). In addition, number of trophallaxis events per bee during a 10min-observation period was counted from the experimental colony while foragers collected 15% w/w sucrose solution for 8 h **(C)**. White boxes represent the reward program number 1 (P1) in which the colony collected unscented 15% w/w sucrose solution. Gray boxes represent the reward program number 2 (P2) in which bees fed for 4 h from a LIO-scented sucrose solution (15% w/w) and afterwards from an unscented solution of equal concentration. The asterisks indicate statistical differences (Panel **A**: ^*^p < 0.05, ^***^p < 0.001, n.s., not significant; Dunn comparison after Kruskal–Wallis test; Panel **B**: ^**^p < 0.01; *G*-test; Panel **C**: ^**^p < 0.01; Mann–Whitney test. After Ramírez et al., 2010. With permission).

In a second experiment performed in queen right colonies, Ramírez et al. (2010) showed that 6/9- and 12/16-day-old bees were able to increase their responsiveness 8 h after a controlled influx of scented food (Figure 1A). Concomitantly with changes in GRSs, olfactory memories to the incoming odor were clearly detected in 12/16-day-old bees over the 24 h period, but not in younger bees (Figure 1B). Interestingly gustatory responsiveness did not change in foragers (Figure 1A), indicating that not all the age groups respond equally to variations in chemosensory information. One possibility is that variations among bees of different ages were related to the currently activity the workers perform. Higher sensitivity to sugar in middle age receivers might play a role during nectar distribution, adjusting the probability of accepting food from incoming foragers according to both gustatory and olfactory nectar characteristics (Pankiw et al., 2004). Lowering sucrose response threshold after memory formation could be thereafter a mechanism that, by increasing the occurrence of mouth-to-mouth food exchanges (trophallaxis) between incoming foragers and food-receiving bees, contributes to the coordination of collective tasks soon after an influx of scented nectar. Higher number of trophallaxis events quantified 4 h after a controlled influx of scented food into the hive (Figure 1C) supports this hypothesis.

## Changes in memory retention according to the age: the role of early olfactory experiences and their consequences on later memory formation

Prior studies described young honeybees as poor learners because they did not perform consistently under laboratory conditions until they were 6/7 days of age (Ray and Ferneyhough, 1997; Morgan et al., 1998; Ichikawa and Sasaki, 2003). Recent reports however, have shown that the honeybee behavior is more plastic at early ages than first thought (Arenas and Farina, 2008; Behrends and Scheiner, 2009). It is assumed that the poor learning performance in newly emerged honeybees is related to the ongoing development of the antennal lobe (AL), which is an important neuropile for olfactory information processing of the bee brain learning (Masson and Arnold, 1984; Morgan et al., 1998). Although part of the honeybee central nervous system involved in olfaction is fully innervated at 2 days adult emergence (Masson et al., 1993), it is believed that the formation of neural circuits concerned in olfactory learning is activity-dependent and young bees need to be subjected to a range of chemosensory stimuli to achieve good learning and memory abilities (Winnington et al., 1996; Farris et al., 2001; Maleszka and Helliwell, 2001; Ichikawa and Sasaki, 2003). Sigg et al. (1997) showed that volumetric increases of the AL glomeruli temporally correlate with activity-dependent improvement in learning performance. In this sense, Brown et al. (2004) showed that changes in the AL are dependent on the performance of foraging activities as the induction of precocious foraging behavior leads to significant increases in both the volume and the number of synapses in this olfactory processing center. Active-dependent maturation is further supported by responses measured in peripheral nervous system. Electrophysiological responses of olfactory antennal receptors increase steadily since emergence up to 4 days of age and remain high until 8 days of adult life (Masson and Arnold, 1987), point where the response to odors decreases if the bees are olfactory deprived (Masson and Arnold, 1984).

More evidence highlighting the plasticity at pre-foraging ages comes from experiments that measured the effect of early olfactory learning later in life (Arenas and Farina, 2008). These experiments showed that associative odor memories established as early as a few days after emergence can be retrieved when bees achieve foraging ages (17 days old; Arenas and Farina, 2008). Furthermore, the same study showed that retention of odor memories is not time-dependent and the learning events that occurred between 5–8 days of adult bees resulted in better olfactory retention than the same learning events occurring before (1–4 days old) or even after (9–12 days old) this period. Such an age-dependent effect of early learning could be observed in bees reared under laboratory conditions, where an odor diluted in the food for 4 consecutive days was the only “floral” odorant source the bees perceived in their whole lives. On the contrary, differences between age groups could not be seen in individuals reared inside the hive (i.e., memories established at both 5–8 and 9–12 days of age were equally well retrieved). Patterns of memory retention depending on the timing the experience took place and the rearing conditions (incubator vs. hive) emphasize the complex interplay between the age of acquisition and the environment during the development of the olfactory pathway.

**Figure 2:**
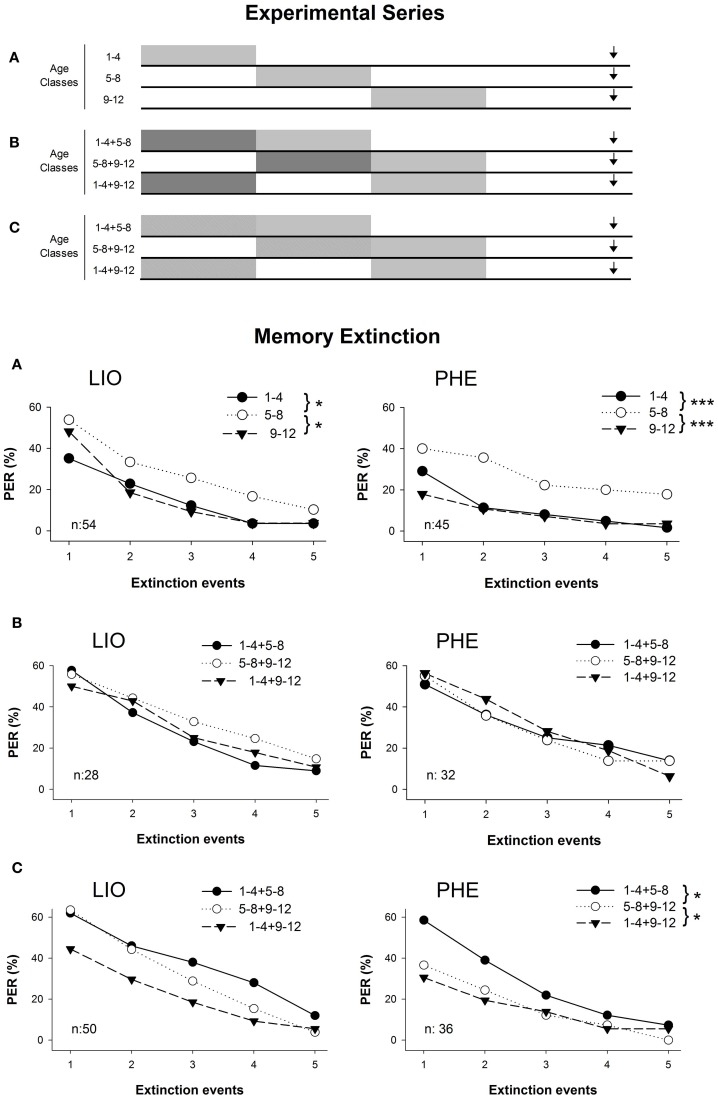
**Extinction response of early olfactory memories during five testing events in the proboscis extension response (PER).** Schedules along the adult lifespan were indicated above for each experimental series. **(A)** Caged bees were offered a scented sugar solution for four consecutive days (gray boxes), and their olfactory memories evaluated at 17 days of age (black arrow). PER to LIO (left panel) or PHE (right panel) were tested when they were offered alone in the sugar solution. **(B)** In addition to the scented solution received for four consecutive days at 5–8 and 9–12 days of age (gray boxes, see **A**), an alternative scented food was previously offered (1–4 or 5–8 days old, dark gray boxes). As result three different treatments were obtained: 1–4 + 5–8, 1–4 + 9–12, and 5–8 + 9–12. **(C)** An odor was exposed as volatile compound for four consecutive days (crossed boxes) before caged bees were offered the scented sugar solution (gray boxes). Whenever LIO was used as the rewarded odor, PHE was used as the non-rewarded or exposed one and vice versa. The asterisks indicate statistical differences between age classes (^***^p < 0.001, ^*^p < 0.05; *post-doc* comparison after RM-ANOVA test). The number of subjects within each experimental series was balanced and the number per treatment is indicated on each graph. (After Arenas et al., 2009a. With permission).

Because consolidation of olfactory memories established at 5–8 days of age might take place through changes that modify structure-function relations when the olfactory system finally matures (Masson and Arnold, 1987; Masson et al., 1993; Winnington et al., 1996; Farris et al., 2001), early olfactory experiences could be important for the complete maturation of the neural pathways. To test whether olfactory memories established later in life are better retrieved if the honeybees have been previously exposed to an early olfactory stimulation, memories established in 5–8 or 9/12-day-old bees were tested after the exposure to a rewarded or unrewarded experience (Arenas et al., 2009a). Briefly, memories established at 5–8 or 9–12 days of age (by means of the offering of scented food, for details see Arenas and Farina, 2008; Figure 2A) were contrasted against those obtained in bees that, in addition to the latter experience, had been subjected to the offering of a second and different scented food at 1–4 or 5–8 days of age (Figure 2B), or exposed to a pure volatile compound delivered in the rearing environment (Figure 2C). Memories quantified in the PER-paradigm by the repeated presentation of the conditioned stimulus (CS) without reinforcement (i.e., 5-trials extinction test; Garelick and Storm, 2005) differed according to the timing and the nature of the prior sensory input (Figure 2). Memories recorded in bees pre-exposed to the rewarded olfactory input (Figure 2B) differed from those obtained in single-odor exposure bees (Figure 2A). These results indicate that early experiences either at the first 4 days of adulthood or at 5–8 days of age clearly enhanced the level of retention of odor-rewarded memories established later in life (at 5–8 or 9–12 days of age). Interestingly, memories established at 9–12 days of age were also improved by the pre-exposure of volatiles in the rearing environment, though its effect was weaker than the one found for the odor-rewarded experiences (Figure 2C). Results coming from rewarded and non-rewarded experiences that precede associative learning showed that relatively brief olfactory stimulations at the early stages of the adult bee's lifespan improve the memorizing process of new learning events.

Together, evidence suggests that young worker bees need to be subjected to the input of chemosensory stimuli, like odors in the food or in the rearing environment, to achieve proper associative learning and memory retention at older ages. Within the hive, bees are constantly exposed to diverse scents and young bees may have the chance to learn odors whilst performing tasks such as nursing or food processing. Thus, learning processes along the in-hive period might prepare workers for later tasks, including those such as foraging, which require the integration of complex cognitive abilities.

## Morphological and functional plasticity of foraging age bees with early odor-rewarded experiences

**Figure 3:**
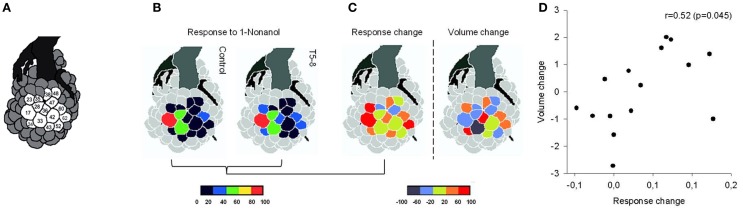
**Morphological and functional glomerular plasticity in 15 identified glomeruli of the honeybee antennal lobe (AL). (A)** Schematic representation of the honeybee antennal lobe (AL) showing the 15 identified glomeruli of the AL labeled with their numbers. **(B)** Glomerular maps of calcium activity in response to 1-Nonanol in 17-day-old control bees (left) and in T5–8 bees (right), that were offered scented food 5–8 days after emergence. **(C)** Change in odor-evoked activity after early olfactory experience (left) and volume change obtained between control and experienced bees (right). Response intensity and volume changes were categorized in five equal bins from 0 to 100% or –100 to 100% respect (see Galizia et al., 1999; Hourcade et al., 2009). **(D)** Structural and functional changes were positively correlated after a glomerulus-wise Pearson correlation (*r* = 0.52, *P* = 0.045, df = 13). (After Arenas et al. 2012. With permission).

The AL (Figure 3) is the primary integrative center of odor information in the insect olfactory system. It is constituted of spherical subunits, the glomeruli, where olfactory receptor neurons from the antennae synapse with local interneurons and second-order neurons connecting with multimodal processing centers such as the mushroom bodies. Odors sensed by olfactory receptors are coded in the AL by patterns of glomerular activity (Friedrich and Korsching, 1997; Joerges et al., 1997; Galizia et al., 1998, 1999; Rubin and Katz, 1999; Sachse et al., 1999; Uchida et al., 2000; Carlsson et al., 2002; Sachse and Galizia, 2002). The arrangement and number of glomeruli that result activated by a particular odor is very well-conserved across adult honeybees; however, this neural code is dynamic and activity patterns can result modified by experience with odors (Faber et al., 1999; Sandoz et al., 2003; Rath et al., 2011).

During the last decade there has been a growing interest in using optical imaging techniques to explore odor-evoked neural activity in the ALs of insects (Joerges et al., 1997; Galizia et al., 1998, 1999; Sachse et al., 1999; Carlsson et al., 2002; Sachse and Galizia, 2002). Using this technique, Wang et al. (2005) recorded neural activity in honeybees of different ages. They showed that odor-evoked neural activity already occurs in the ALs of individuals as young as 1 or 2 days of age. Despite the relatively weak responses to odors in young bees, glomerular activity patterns were odor-specific, suggesting that the neural substrate for odor representation is already set up before emergence.

To study the changes induced by early associative learning events on the functional and structural properties of adult neural networks of the honeybee, the activity in the AL of 17-day-old honeybees which have experienced 1-Nonanol (1-NON) diluted in sucrose solution 5–8 days after emergence were recorded (Arenas et al., 2009b). This study showed that the conditioned odor evokes enhanced glomerular activity and modifies spatiotemporal response patterns. Figure 3 shows how the glomerular maps of calcium responses evoked by 1-NON in control bees (naïve) and in bees subjected to early learning (T5–8) look like. Differences between the response patterns between these two groups are presented in Figure 3B. Map of relative response change shows the additional activation of glomeruli 23, 24, 36, and 62 as the main variation induced in learned bees (Figures 3A–C).

To investigate whether this reorganization translates into structural changes within the AL, the volume of 15 identified glomeruli in control and T5–8 bees, which had established memories with 1-Hexanol or 1-NON were also measured (Arenas et al., 2012). By comparing data from treated bees to bees without such experience, we showed that early olfactory learning results in the AL structural modifications (i.e., glomerular volume variations). Increases in glomerular volume appeared to be specific to the learned odor as 1-Hexanol and 1-NON long-term memories-induced changes in selective sets of glomeruli.

Comparison between volumetric measures and functional modifications in the AL network (i.e., calcium-imaging recordings) determined that those glomeruli showing structural changes after early learning were those that exhibited a significant increase in neural activity. Map of volume change in bees subjected to early learning events with 1-NON shows that the four newly recruited glomeruli (23, 24, 36, and 62) were those that also exhibited the largest volume increases (Figure 3B). Moreover, the hypothesis that glomerular volume changes took place in the same set of glomeruli that change the most in their odor-evoked activity was consistent with the glomerulus-wise correlation found between structural and functional changes (Figure 3C).

These results indicated that early olfactory learning results in long-lasting structural and functional modifications of the AL network in the form of glomerular volume variations and on the activation of new glomeruli upon olfactory stimulation with the odor that has been learned. It is then showed that early odor-rewarded experience induces a stable reorganization of olfactory circuits that accompanies the high plasticity of behavior, presumably because the olfactory system finishes its maturation at that stage (Masson and Arnold, 1987; Winnington et al., 1996). This study demonstrated that the AL is a site in which both structural and functional plasticity can be observed following the formation of long-term olfactory memories (Grünbaum and Müller, 1998; Müller, 2000; Hourcade et al., 2009).

## Early odor-rewarded experiences on social life and their effects on recruitment

Successful foragers perform waggle dances to communicate hive mates the location of the site they are visiting (von Frisch, 1967). In addition to spatial information, following bees can learn the odors of the exploited food source when small samples of food are shared via trophallaxis between the dancing bee and the follower during the short interruptions in-between dance maneuvers (Díaz et al., 2007). Floral scents learned in the recruiting context represent thereafter an important informational cue that assists recruited followers while search for the advertised goal (von Frisch, 1967; Dyer, 2002).

It has been shown that “unemployed” foragers that had visited a scented food source preferred to follow dancers carrying the odors they knew from previous field trips (Grüter et al., 2008). As a consequence, they were more likely to be reactivated to resume foraging tasks (Biesmejer and Seeley, 2005; Grüter et al., 2008). Biases in choice patterns however, may not be restricted to foragers that experienced the scent outside the hive, but may also involve followers that have experienced the floral scents inside the nest many days ago (Farina et al., 2005; Arenas et al., 2008; Grüter et al., 2009).

**Figure 4:**
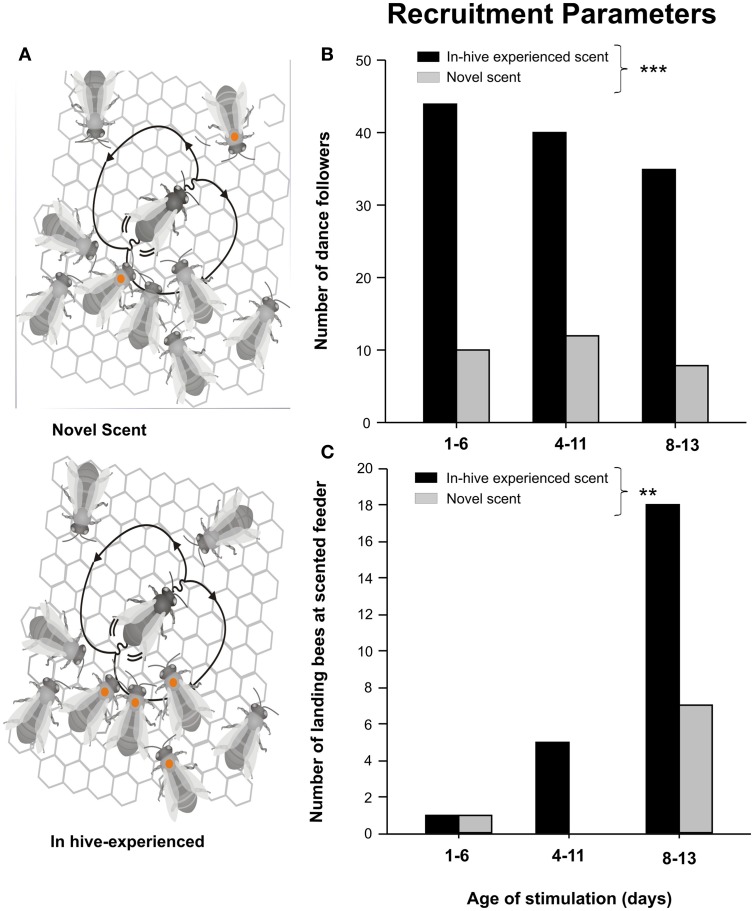
**Biased behavior within the recruiting context. (A)** Schematic representation of the biased following behavior in both experimental situations: novel scent *(above)* and in-hive experienced scent *(below)*. **(B)** Number of dance followers engaged with dancers carrying either the experienced scent or a novel scent introduced 8 days ago by means of foragers. **(C)** Number of arrivals to the foraging location advertised by dancers carrying either the experienced scent or a novel scent. The asterisks indicate statistical differences between situations (number of dance followers: ^***^p < 0.001, after GLMM test; arrivals: ^**^p < 0.01, after Fisher's exact test). (After Balbuena et al., 2012. With permission).

By introducing newly emerged color-marked bees into a glass-wall hive we study whether an influx of scented food offered at pre-foraging ages influence bees' interaction patterns and the chances of being recruited many days later (Balbuena et al., 2012). Eight days after introducing 70 ml of scented food into the hive by means of trained foragers, the number of color-marked bees either engaged in following dancers coming from distant feeders scented with the experienced odor or scented with a novel odor was quantified (Figure 4A). Bees that experienced the odor at 1–6, 4–11, and 8–13 days of age showed a stronger bias toward following the dancers carrying the experienced scent than with dancers carrying the novel scent (Figure 4B). Concomitantly with the increase in the number of bees following these dancers, more color-marked bees arriving at the feeders characterized with the experienced scent were observed (Balbuena et al., 2012; Figure 4C). Then, alteration in the patterns of interaction during dances suggests that previous experiences with the odor impact the chances of followers to be recruited by dancers that carried the experienced scent.

Preferences in the choice of dancing partners and biases in recruitment might be explained by stable and long-term olfactory memories acquired inside the hive during the influx of scented food 8 days ago (Arenas et al., 2007, 2008; Arenas and Farina, 2008; Grüter et al., 2009). The idea that dance followers acquired floral scent information while performing in-hive (non-foraging-related) tasks influenced recruitment on a long-term scale is consistent with the retrieval of olfactory memories established early in life and tested at foraging ages (Arenas and Farina, 2008; Grüter et al., 2009). Therefore, these results provide evidence that food-related information acquired by honeybees while performing in-hive tasks is functional (and putatively adaptive) in the recruiting context by facilitating the decoding of the spatial information transmitted in the waggle dance.

## Remarks and conclusions

A honeybee colony can forage several floral species simultaneously when available. Thus, each successful foraging bee brings back different types of nectar (differing in smell and taste) to the hive. Within this complex chemical environment, middle age workers involved in food reception can learn food odors through mouth-to-mouth trophallaxis while they unload and store the scented food. One-week-old bees performing tasks such as nursing have similar opportunities when handle scented food directly from comb cells to feed the brood (Winston, 1987).

Although it was suggested that young honeybees did not learn consistently under laboratory conditions until they were 6/7 days of age (Ray and Ferneyhough, 1997; Morgan et al., 1998; Ichikawa and Sasaki, 2003), today there is enough evidences supporting that olfactory experiences gained soon after emergence are important to achieve proper learning and the abilities to form memories (Arenas and Farina, 2008; Arenas et al., 2009a; Grüter et al., 2009). Tuning the olfactory system by means of different rewarded and unrewarded odor inputs might prepare workers to face more complex tasks later in life. Consolidation of early odor memories (i.e., established at 5–8 days of age) may take place through changes that modify the structure and the function of the AL (first neuropile that process odor information), by the time the nervous system involving in olfaction goes through its final steps of maturation (Masson and Arnold, 1987; Winnington et al., 1996).

At the individual and social level the presence of long-lasting odor information might have important consequences during the resource exploitation. The novice foragers might be prompted to search for sources whose scents are reminiscent of the odors learned at early ages. Furthermore, retrieval of early olfactory memories may participate in the coordination of collective tasks, leading to non-random interactions between foragers and experienced followers within the dancing context. Biases in the choice of dancing partners favor recruitment of foragers that despite naïve for the advertised food source, known in advance how it smells.

Floral odorant cues also alter how early and middle age bees perceive and respond to nectars of different qualities. Given the fact that the speed and extent the information propagates amongst nest mates rest on the quality and odorant cues of the food (Ramírez et al., 2010; Farina et al., 2012), individuals with a high plasticity in their response to changes in nectar characteristic have profound effects on the overall balance between foraging and processing capacity of the colony. In changing environments, accurate modulation of sensory-response systems in bees in charge of linking out and in-hive duties seems to be important to ensure the incoming of the best quality nectars available in the surrounding. To what extent variations in chemosensory information are triggered by associative learning in young adult bees remains a subject to study.

Abundance and composition of flower species have profound ecological consequences on pollinators since both can change over their short lifespan (Kearns and Inouye, 1993; Willmer and Stone, 2004). As long as rewarding floral types remain stable for a few weeks, early experienced bee would enhance the chances of the colony of foraging accurately and efficiently on these alternatives. Once the floral market changes and the odors precociously learned are no longer available by the time the bees initiate their foraging-related activities, the extraordinary neural plasticity of bees (Menzel, 1999, 2012; Giurfa, 2007) would allow adjusting their foraging preferences according to new and more profitable opportunities.

### Conflict of interest statement

The authors declare that the research was conducted in the absence of any commercial or financial relationships that could be construed as a potential conflict of interest.

## References

[B1] Arenas A. Farina W. M. (2008). Age and rearing environment interact in the retention of early olfactory memories in honeybees. J. Comp. Physiol. A 194, 629–640 10.1007/s00359-008-0337-z 18438671

[B2] Arenas A. Fernández V. M. Farina W. M. (2007). Floral odor learning within the hive affects honeybees' foraging decisions. Naturwissenschaften 94, 218–222 10.1007/s00114-006-0176-0 17119909

[B3] Arenas A. Fernández V. M. Farina W. M. (2008). Floral scents experienced within the colony affect long-term foraging preferences in honeybees. Apidologie 39, 714–722

[B4] Arenas A. Fernández V. M. Farina W. M. (2009a). Associative learning during early adulthood enhances later memory retention in honeybees. PLoS ONE 4:e8046 10.1371/journal.pone.0008046 19956575 PMC2779852

[B4a] Arenas A. Giurfa M. Farina W. M. Sandoz J. C. (2009b). Early olfactory experience modifies neural activity in the antennal lobe of a social insect at the adult stage. Eur. J. Neurosci. 30, 1498–1508 10.1111/j.1460-9568.2009.06940.x 19821839

[B4b] Arenas A. Giurfa M. Sandoz J. C. Hourcade B. Devaud J. M. Farina W. M. (2012). Early olfactory experience induces structural changes in the primary olfactory center of an insect brain. Eur. J. Neurosci. 35, 682–690 10.1111/j.1460-9568.2012.07999.x 22300014

[B5] Balbuena M. S. Arenas A. Farina W. M. (2012). Floral scents learned inside the honeybee hive have a long-lasting effect on recruitment. Anim. Behav. 84, 77–83

[B7] Behrends A. Scheiner R. (2009). Evidence for associative learning in newly emerged honey bees (*Apis mellifera*). Anim. Cogn. 12, 249–255 10.1007/s10071-008-0187-7 18791751

[B8] Biesmejer J. C. Seeley T. D. (2005). The use of waggle dance information by honey bees throughout their foraging careers. Behav. Ecol. Sociobiol. 59, 133–142

[B9] Bitterman M. E. Menzel R. Fietz A. Schäfer S. (1983). Classical conditioning of proboscis extension in honeybees (*Apis mellifera*). J. Comp. Psychol. 97, 107–119 6872507

[B10] Brown S. M. Napper R. M. Mercer A. R. (2004). Foraging experience, glomerulus volume, and synapse number: a stereological study of the honey bee antennal lobe. J. Neurobiol. 60, 40–50 10.1002/neu.20002 15188271

[B11] Carlsson M. A. Galizia C. G. Hansson B. S. (2002). Spatial representation of odours in the antennal lobe of the moth *Spodoptera littoralis* (Lepidoptera: Noctuidae). Chem. Senses 27, 231–244 10.1093/chemse/27.3.231 11923186

[B12] Cornwell-Jones C. A. Velasquez P. Wright E. L. McGaugh J. L. (1988). Early experience influences adult retention of aversively motivated tasks in normal, but not DSP4-treated rats. Dev. Psychobiol. 21, 177–185 10.1002/dev.420210206 3345869

[B13] Cramer C. P. Pfister J. P. Haig K. A. (1988). Experience during suckling alters later spatial learning. Dev. Psychobiol. 21, 1–24 10.1002/dev.420210102 3338625

[B14] Díaz P. C. Grüter C. Farina W. M. (2007). Floral scents affect the distribution of hive bees around dancers. Behav. Ecol. Sociobiol. 61, 1589–1597

[B15] Dukas R. (2008). Evolutionary biology of insect learning. Annu. Rev. Entomol. 53, 145–160 10.1146/annurev.ento.53.103106.093343 17803459

[B16] Dyer F. C. (2002). The biology of the dance language. Annu. Rev. Entomol. 47, 917–949 10.1146/annurev.ento.47.091201.145306 11729095

[B17] Faber T. Joerges J. Menzel R. (1999). Associative learning modifies neural representations of odors in the insect brain. Nat. Neurosci. 2, 74–78 10.1038/4576 10195183

[B18] Fahrbach S. E. (2006). Structure of the mushroom bodies of the insect brain. Annu. Rev. Entomol. 51, 209–232 10.1146/annurev.ento.51.110104.150954 16332210

[B19] Farina W. M. Grüter C. Arenas A. (2012). Olfactory information transfer during recruitment in honey bees, in Honeybee Neurobiology and Behavior: A Tribute to Randolf Menzel, eds Galizia C. G. Eisenhardt D. Giurfa M. (Dordrecht; Heidelberg; London; New York: Springer), 89–102

[B20] Farina W. M. Grüter C. Díaz P. C. (2005). Social learning of floral odours inside the honeybee hive. Proc. Biol. Sci. 272, 1923–1928 10.1098/rspb.2005.3172 16191598 PMC1559887

[B21] Farris S. M. Robinson G. E. Fahrbach S. E. (2001). Experience –and age- related outgrowth of intrinsic neurons in the mushroom bodies of the adult worker honeybee. J. Neurosci. 21, 6395–6404 11487663 10.1523/JNEUROSCI.21-16-06395.2001 PMC6763189

[B22] Friedrich R. W. Korsching S. I. (1997). Combinatorial and chemotopic odorant coding in the zebrafish olfactory bulb visualized by optical imaging. Neuron 18, 737–752 10.1016/S0896-6273(00)80314-1 9182799

[B23] Galizia C. G. Franke T. Menzel R. Sandoz J. C. (2012). Optical imaging of concealed brain activity using a gold mirror in honeybees. J. Insect Physiol. 55, 743–749 10.1016/j.jinsphys.2012.02.010 22414536

[B24] Galizia C. G. Nägler K. Hölldobler B. Menzel R. (1998). Odour coding is bilaterally symmetrical in the AL of honeybees (*Apis mellife*ra). Eur. J. Neurosci. 10, 2964–2974 10.1111/j.1460-9568.1998.00303.x 9758166

[B25] Galizia C. G. Sachse S. Rappert A. Menzel R. (1999). The glomerular code for odor representation is species specific in the honeybee *Apis mellifera*. Nat. Neurosci. 2, 473–478 10.1038/8144 10321253

[B26] Garelick M. G. Storm D. R. (2005). The relationship between memory retrieval and memory extinction. Proc. Natl. Acad. Sci. U.S.A. 102, 9091–9092 10.1073/pnas.0504017102 15967979 PMC1166639

[B28] Gerber B. Geberzahn N. Hellstern F. Klein J. Kowalksy O. Wüstenberg D. (1996). Honey bees transfer olfactory memories established during flower visits to a proboscis extension paradigm in the laboratory. Anim. Behav. 52, 1079–1085

[B29] Giurfa M. (2007). Behavioral and neural analysis of associative learning in the honeybee: a taste from the magic well. J. Comp. Physiol. A 193, 801–824 10.1007/s00359-007-0235-9 17639413

[B30] Giurfa M. Sandoz (2012). Invertebrate learning and memory: fifty years of olfactory conditioning of the proboscis extension response in honeybees. Learn. Mem. 19, 54–66 10.1101/lm.024711.111 22251890

[B31] Gerber B. Geberzahn N. Hellstern F. Klein J. Kowalksy O. Wustenberg D. (1996). Honey bees transfer olfactory memories established during flower visits to a proboscis extension paradigm in the laboratory. Anim. Behav. 52, 1079–1085

[B33] Grünbaum L. Müller U. (1998). Induction of a specific olfactory memory leads to a long-lasting activation of protein kinase C in the antennal lobe of the honeybee. J. Neurosci. 18, 4384–4392 9592115 10.1523/JNEUROSCI.18-11-04384.1998 PMC6792810

[B34] Grüter C. Acosta L. E. Farina W. M. (2006). Propagation of olfactory information within the honeybee hive. Behav. Ecol. Sociobiol. 60, 707–715

[B35] Grüter C. Balbuena M. S. Farina W. M. (2008). Informational conflicts created by the waggle dance. Proc. Biol. Sci. 275, 1321–1327 10.1098/rspb.2008.0186 18331980 PMC2602683

[B36] Grüter C. Balbuena M. S. Farina W. M. (2009). Retention of long-term memories in different age groups of honeybee (*Apis mellifera*) workers. Ins. Soc. 56, 385–387

[B37] Grüter C. Farina W. M. (2009). The honeybee waggle dance: can we follow the steps? Trends Ecol. Evol. 24, 242–247 10.1016/j.tree.2008.12.007 19307042

[B38] Gschanes A. Eggenreich U. Windisch M. Crailsheim K. (1998). Early postnatal stimulation influences passive avoidance behaviour of adult rats. Behav. Brain Res. 93, 91–98 9659991 10.1016/s0166-4328(97)00143-5

[B39] Hourcade B. Perisse E. Devaud J. M. Sandoz J. C. (2009). Long-term memory shapes the primary olfactory center of an insect brain. Learn. Mem. 16, 607–615 10.1101/lm.1445609 19794186

[B40] Ichikawa N. Sasaki M. (2003). Importance of social stimuli for the development of learning capability in honeybee. Appl. Entomol. Zool. 38, 203–209

[B41] Joerges J. Küttner A. Galizia C. G. Menzel R. (1997). Representations of odors and odor mixtures visualized in the honeybee brain. Nature 387, 285–287

[B42] Kearns C. A. Inouye D. W. (1993). Techniques for Pollination Biologists. Boulder, CO: University Press of Colorado

[B43] Kuwabara M. (1957). Bildung Des Bedingten Reflexes Von Pavlovs Typus Bei Der Honigbiene, Apis Mellifera. Fukuoka: Biologisches Institut, Naturwissenschaftliche Fakultät, Universität Kyushu

[B44] Lindauer M. (1952). Ein Beitrag zur Frage der Arbeitsteinlung im Bienenstaat. Z vergl Physiol. 34, 299–345

[B45] Lorenz K. (1935). Der Kumpan in der Umwelt des Vogels. Zeitschrift für Ornithologie 83, 137–213, 289–413.

[B46] Maleszka R. Helliwell P. (2001). Effect of juvenile hormone on short-term olfactory memory in young honeybees (*Apis mellifera*) Horm. Behav. 40, 403–408 10.1006/hbeh.2001.1705 11673913

[B47] Martinez A. Farina W. M. (2008). Honeybees modify gustatory responsiveness after receiving nectar from foragers within the hive. Behav. Ecol. Sociobiol. 62, 529–535 10.1007/s00114-006-0157-3 17021915

[B48] Masson C. Arnold G. (1984). Ontogeny, maturation and plasticity of the olfactory system in the worker bee. J. Insect Physiol. 30, 7–14 9578445

[B49] Masson C. Arnold G. (1987). Organization and plasticity of the olfactory system of the honeybee, *Apis mellifera*, in Neurobiology and Behavior of Honeybee, eds Menzel R. Mercer A. (Berlin, Heidelberg: Springer Verlag), 280–295

[B50] Masson C. Pham-Delègue M. H. Fonta C. Gascuel J. Arnold G. Nicolas G. (1993). Recent advances in the concept of adaptation to natural odour signals in the honeybee *Apis mellifera* L. Apidologie 24, 169–194

[B51] Matthews K. Robbins T. W. (2003). Early experience as a determinant of adult behavioral responses to reward: the effects of repeated maternal separation in the rat. Neurosci. Biobehav. Rev. 27, 45–55 10.1016/S0149-7634(03)00008-3 12732222

[B52] Menzel R. (1999). Memory dynamics in the honeybee. J. Comp. Physiol. A 185, 323–340

[B53] Menzel R. (2012). The honeybee as a model for understanding the basis of cognition. Nature 13, 758–768 10.1038/nrn3357 23080415

[B55] Michener C. D. (1974). The Social Behavior of Bees: A Comparative Study. Cambridge: Harvard University Press

[B56] Morgan S. M. Butz Huryn V. M. Downes S. R. Mercer A. R. (1998). The effects of queenlessness on the maturation of the honey bee olfactory system. Behav. Brain Res. 91, 115–126 10.1016/S0166-4328(97)00118-6 9578445

[B57] Müller U. (2000). Prolonged activation of cAMP-dependent protein kinase during conditioning induces long-term memory in honeybees. Neuron 27, 159–168 10.1016/S0896-6273(00)00017-9 10939339

[B58] Neal W. R. (1972). The effect of environmental deprivation on speech and language development: Implications for child care workers. Child Care Q. 1, 157–172

[B59] Page R. E. Erber J. Fondrk M. K. (1998). The effect of genotype on response thresholds to sucrose and foraging behavior of honey bees (*Apis mellifera* L.). J. Comp. Physiol. A 182, 489–500 10.1007/s003590050196 9565478

[B60] Pankiw T. Page R. E. Jr. (1999). The effect of genotype, age, sex, and caste on response thresholds to sucrose and foraging behavior of honey bees (*Apis mellifera* L.). J. Comp. Physiol. A 185, 207–213 10.1007/s003590050379 10488557

[B61] Pankiw T. Nelson M. Page R. E. Fondrk M. K. (2004). The communal crop: modulation of sucrose response thresholds of pre-foraging honey bees with incoming nectar quality. Behav. Ecol. Sociobiol. 55, 286–292

[B62] Pankiw T. Waddington K. D. Pager R. E. Jr. (2001). Modulation of sucrose response thresholds in honey bees (*Apis mellifera* L.): influence of genotype, feeding, and foraging experience. J. Comp. Physiol. A 187, 293–301 11467502 10.1007/s003590100201

[B63] Pryce C. R. Feldon J. (2003). Long-term neurobehavioural impact of the postnatal environment in rats: manipulations, effects and mediating mechanisms. Neurosci. Biobehav. Rev. 27, 57–71 10.1016/S0149-7634(03)00009-5 12732223

[B64] Ramírez G. P. Martínez A. S. Fernández V. M. Corti Bielsa G. Farina W. M. (2010). The influence of gustatory and olfactory experiences on responsiveness to reward in the honeybee. PLoS ONE 5:e13498 10.1371/journal.pone.0013498 20975953 PMC2958144

[B65] Rath L. Galizia G. Szsyszka P. (2011). Multiple memory traces after associative learning in the honey bee antennal lobe. Eur. J. Neurosci. 34, 352–360 10.1111/j.1460-9568.2011.07753.x 21692886

[B66] Ray S. Ferneyhough B. (1997). The effects of age on olfactory learning and memory in the honey bee *Apis mellifera*. Learn. Mem. 8, 789–793 9106768 10.1097/00001756-199702100-00042

[B67] Riley J. R. Greggers U. Smith A. D. Reynolds D. R. Menzel R. (2005). The flight paths of honeybees recruited by the waggle dance. Nature 435, 205–207 10.1038/nature03526 15889092

[B68] Robinson G. (1992). Regulation of division of labor in insect societies. Annu. Rev. Entomol. 37, 637–665 10.1146/annurev.en.37.010192.003225 1539941

[B69] Rösch G. A. (1925). Untersuchungen uber die Arbeitsteilung im Bienenstaat. 1. Teil: Die Tatigkeiten im normalen Bienenstaate und ihre Beziehungen zum Alter der Arbeitsbienen. Z vergl Physiol. 2, 571–631

[B70] Rubin B. D. Katz L. C. (1999). Optical imaging of odorant representations in the mammalian olfactory bulb. Neuron 23, 499–511 10.1016/S0896-6273(00)80803-X 10433262

[B71] Sachse S. Galizia C. G. (2002). Role of inhibition for temporal and spatial odor representation in olfactory output neurons: a calcium imaging study. J. Neurophysiol. 87, 1106–1117 11826074 10.1152/jn.00325.2001

[B72] Sachse S. Rappert A. Galizia C. G. (1999). The spatial representation of chemical structures in the AL of honeybees: steps towards the olfactory code. Eur. J. Neurosci. 11, 3970–3982 10.1046/j.1460-9568.1999.00826.x 10583486

[B73] Sandoz J. C. Galizia C. G. Menzel R. (2003). Side-specific olfactory conditioning leads to more specific odor representation between sides but not within sides in the honeybee antenal lobe. Neuroscience 120, 1137–1148 10.1016/S0306-4522(03)00384-1 12927218

[B74] Sandoz J. C. Laloi D. Odoux J. F. Pham-Delegué M. H. (2000). Olfactory information transfer in the honeybee: compared efficiency of classical conditioning and early exposure. Anim. Behav. 59, 1025–1034 10.1006/anbe.2000.1395 10860530

[B75] Schäble S. Poeggel G. Braun K. Gruss M. (2007). Long-term consequences of early experience on adult avoidance learning in female rats: role of the dopaminergic system. Neurobiol. Learn. Mem. 87, 109–122 10.1016/j.nlm.2006.07.005 16938473

[B76] Scheiner R. Erber J. Page R. E. Jr. (1999). Tactile learning and the individual evaluation of the reward in honey bees (*Apis mellifera* L.). J. Comp. Physiol. A 185, 1–10 10.1007/s003590050360 10450609

[B77] Scheiner R. Page R. E. Jr. Erber J. (2001). The effects of genotype, foraging Role, and sucrose responsiveness on the tactile learning performance of honey bees (*Apis mellifera* L.). Neurobiol. Learn. Mem. 76, 138–150 10.1006/nlme.2000.3996 11502146

[B78] Seeley T. D. (1982). Adaptive significance of the age polyethism schedule in honeybee colonies. Behav. Ecol. Sociobiol. 11, 287–293

[B79] Seeley T. D. (1989). Social foraging in honeybees: how nectar foragers assess their colony's nutritional status. Behav. Ecol. Sociobiol. 24, 181–199 10.1242/jeb.02025 16424092

[B80] Sigg D. Thompson C. M. Mercer A. R. (1997). Activity-dependent changes to the brain and behavior of the honey bee, *Apis mellifera* (L.). J. Neurosci. 17, 7148–7156 9278549 10.1523/JNEUROSCI.17-18-07148.1997 PMC6573286

[B81] Takeda K. (1961). Classical conditioned response in the honey bee. J. Insect Physiol. 6, 168–179

[B82] Uchida N. Takahashi Y. K. Tanifuji M. Mori K. (2000). Odor maps in the mammalian olfactory bulb: domain organization and odorant structural features. Nat. Neurosci. 3, 1035–1043 10.1038/79857 11017177

[B83] von Frisch K. (1967). The Dance Language and Orientation of Bees. Cambridge, MA: Harvard University Press

[B84] Wang W. S. Zhang S. Sato K. Srinivasan M. V. (2005). Maturation of odor representation in the honeybee antennal lobe. J. Insect Physiol. 51, 1244–1254 10.1016/j.jinsphys.2005.07.003 16183074

[B85] Willmer P. G. Stone G. N. (2004). Behavioral, ecological, and physiological determinants of the activity patterns of bees. Adv. Stud. Behav. 34, 347–466

[B86] Wilson E. O. (1971). The Insect Societies. Cambridge, MA: Harvard University Press

[B87] Winnington A. P. Napper R. M. Mercer A. R. (1996). Structural plasticity of identified glomeruli in the antennal lobes of the adult worker honey bee. J. Comp. Neurol. 365, 479–490 10.1002/(SICI)1096-9861(19960212)365:3<479::AID-CNE10>3.0.CO;2-M 8822183

[B88] Winston M. L. (1987). Biology of the Honey Bee. Cambridge: Harvard University Press

